# Non-structural Proteins of Severe Fever With Thrombocytopenia Syndrome Virus Suppress RNA Synthesis in a Transcriptionally Active cDNA-Derived Viral RNA Synthesis System

**DOI:** 10.3389/fmicb.2021.709517

**Published:** 2021-08-16

**Authors:** Fuli Ren, Shu Shen, Yun-Jia Ning, Qiongya Wang, Shiyu Dai, Junming Shi, Min Zhou, Hualin Wang, Chaolin Huang, Ding-Yu Zhang, Fei Deng

**Affiliations:** ^1^Research Center for Translational Medicine, Wuhan Jinyintan Hospital, Wuhan, China; ^2^State Key Laboratory of Virology, Wuhan Institute of Virology, Chinese Academy of Sciences, Wuhan, China; ^3^National Virus Resource Center, Wuhan Institute of Virology, Chinese Academy of Sciences, Wuhan, China; ^4^Department of Infectious Diseases, Wuhan Jinyintan Hospital, Wuhan, China

**Keywords:** bunyavirus, RNA synthesis, HRTV, SFTSV RNA synthesis and regulation, NSs, SFTSV, minigenome

## Abstract

Severe fever with thrombocytopenia syndrome (SFTS) is an emerging infectious disease caused by the tick-borne SFTS bunyavirus (SFTSV) resulting in a high fatality rate up to 30%. SFTSV is a negative-strand RNA virus containing three single-stranded RNA genome segments designated as L, M, and S, which respectively, encode the RNA-dependent RNA polymerase (RdRp), glycoproteins Gn and Gc, and nucleoprotein (N) and non-structural proteins (NSs). NSs can form inclusion bodies (IBs) in infected and transfected cells. A previous study has provided a clue that SFTSV NSs may be involved in virus-like or viral RNA synthesis; however, the details remain unclear. Our work described here reveals that SFTSV NSs can downregulate virus-like RNA synthesis in a dose-dependent manner within a cDNA-derived viral RNA synthesis system, i.e., minigenome (−) and minigenome (+) systems based on transfection, superinfection, and luciferase reporter activity determination; meanwhile, NSs also show a weak inhibitory effect on virus replication. By using co-immunoprecipitation (Co-IP) and RT-PCR combined with site-directed mutagenesis, we found that NSs suppress virus-like RNA or virus replication through interacting with N but not with RdRp, and the negative regulatory effect correlates closely with the IB structure it formed but is not associated with its role of antagonizing host innate immune responses. When the cytoplasmic structure of IB formed by SFTSV NSs was deprived, the inhibitory effect of NSs on virus-like RNA synthesis would weaken and even disappear. Similarly, we also evaluated other bandavirus NSs that cannot form IB in neither infected nor transfected cells, and the results showed that the NSs of Heartland bandavirus (HRTV) did not show a significant inhibitory effect on virus-like RNA synthesis within a minigenome system. Our findings provide experimental evidence that SFTSV NSs participate in regulating virus-like or viral RNA synthesis and the negative effect may be due to the NSs–N interaction.

## Introduction

Severe fever with thrombocytopenia syndrome virus (SFTSV) is an emerging tick-borne bunyavirus first isolated and identified in 2009 in the rural areas of Henan Province, China (Yu et al., 2011). SFTSV is now classified into the genus *Bandavirus*, family *Phenuiviridae*, and order *Bunyavirales* by the International Committee on Taxonomy of Viruses (ICTV) (Kuhn et al., 2020). In addition, it is the causative pathogen of severe fever with thrombocytopenia syndrome (SFTS) and is mainly prevalent in East Asian countries, including China, Japan, South Korea, and Vietnam (Takahashi et al., 2014; Kim et al., 2018; Tran et al., 2019). SFTS is mainly characterized by thrombocytopenia syndrome (Yu et al., 2011; Lei et al., 2015), and in some severe patients, it can lead to multiple organ failure or even death (Li et al., 2018; Song et al., 2018), resulting in high fatality rates varying from 12 to 30% in different areas (Park et al., 2019). However, there is no available vaccine or therapeutic drugs for SFTSV. Recently, several tick-borne bunyavirus related to SFTSV, including HRTV and Guertu virus (GTV), have been isolated and identified in United States and China, respectively (McMullan et al., 2012; Shen et al., 2018; Staples et al., 2020). Although the pathogenicity of GTV to human remains unclear, patients with infection of HRTV presented very similar clinical symptoms to SFTS patients. Our previous data showed that SFTSV, HRTV, and even GTV have the potential to undergo genome reassortment (Ren et al., 2020), which may lead to the generation of unknown progeny viruses, thus highlighting growing public health threat posed by bandaviruses.

In segmented negative-strand RNA genome viruses, genomic and antigenomic viral RNA (vRNA and cRNA) but not mRNA are always found assembled with multiple copies of a nucleoprotein (N) into nucleocapsids (Mir and Panganiban, 2006; Reguera et al., 2014; Te Velthuis and Fodor, 2016; Sun et al., 2018). During viral replication, the genomic RNA undergoes encapsidation, and the coated viral genomic RNAs assemble with the viral polymerase (RdRp) to form RNPs (vRNP), which are central to the viral life cycle and can be packaged into progeny virus particles (Hornak et al., 2016; Wichgers Schreur et al., 2018). Then, the viral polymerase RdRp synthesizes full-length antigenomic RNA (cRNA) using vRNA as template in a primer-independent manner, and cRNA subsequently can be used as template for the synthesis of progeny genomic RNA (vRNA) (Reguera et al., 2016). Meanwhile, viral mRNA encoding viral structural and non-structural proteins (NSs) (e.g., RdRp, glycoprotein Gn, Gc, N, and NSs) can be synthesized through the so-called secondary transcription by using newly synthesized vRNA as templates. SFTSV RdRp and N are both implicated in viral RNA synthesis and play indispensable roles (Kolakofsky and Hacker, 1991; Guu et al., 2012; Amroun et al., 2017; Sun et al., 2018). However, besides these two proteins, other viral proteins may also participate in the viral RNA synthesis, for example, the NSs.

Unlike L and M segments, the S segment of SFTSV adopts ambisense coding strategies to encode N and NSs. It has been demonstrated that N can form oligomers, such as tetramer, pentamer, and hexamer, and is associated with viral RNA encapsidation (Jiao et al., 2013), an important process for viral RNP formation and RNA synthesis (Dong et al., 2013; Li et al., 2013; Reguera et al., 2013; Zheng and Tao, 2013). Although the NSs of bunyaviruses share poor amino acid similarity, the strategies they utilize to hijack host cells are similar (Ly and Ikegami, 2016). SFTSV NSs mainly distribute in the cytoplasm and can form inclusion bodies (IBs) in virus-infected cells and plasmid-transfected cells, and it can suppress the activities of the beta interferon (IFN-β) promoter by interacting with host kinases TBK1/IKKε (Ning et al., 2014, 2015; Wu et al., 2014). In HRTV bandavirus, the NSs disrupt host defenses by blocking the TBK1 kinase–IRF3 transcription factor interaction and signaling required for interferon induction (Ning et al., 2017). In newly isolated Guertu virus (GTV), the NSs can form IBs and extended filamentous structures (FSs) that can diminish the IFN induction through sequestering TBK1 and STAT2 (Min et al., 2020). Interestingly, Wu et al. (2014) and Brennan et al. (2015) have separately provided clues that SFTSV NSs may also play roles in virus-like or viral RNA synthesis and virus replication; Wu also reported that NSs-formed viroplasm-like structures or IBs co-localize with viral S segment. However, the mechanisms of bandavirus including SFTSV and HRTV NSs participating in and regulating viral RNA synthesis were not fully elucidated as yet.

Effect of virus proteins on cDNA-derived virus-like RNA expression has been shown for several important bunyaviruses utilizing minigenome systems, in which the altered levels of virus-like RNA synthesis can be quantified by using the reporter protein as an indicator. For example, Ikegami et al. (2005) have used the reverse genetics system to demonstrate that the NSs of Rift Valley Fever virus (RVFV) promote viral RNA synthesis in an RVFV T7 RNA polymerase-driven minigenome system; a further study revealed that RVFV NSs lead to a specific degradation of PKR that is responsible for translational arrest of cellular and virus mRNAs (Habjan et al., 2009). Meanwhile, RVFV NSs also inhibit cellular transcription by targeting the cellular TFIIH transcription factor (Le May et al., 2004). In the case of Bunyamwera virus (BUNV), it has been reported that the NSs of BUNV inhibit viral RNA synthesis in a minigenome system (Weber et al., 2001) and host cell transcription (Leonard et al., 2006). Previously, we have established the mouse polymerase I (pol I)-driven minigenome (−) system to dissect the genome reassortment potential between SFTSV and HRTV and to identify the elements located in the UTR affecting viral promoter activity (Ren et al., 2020). Here, we established a minigenome (+) that can generate antigenome cRNA analog; by using the cDNA-derived virus-like RNA synthesis systems including minigenome (−) and minigenome (+) systems, we revealed the involvement of SFTSV NSs in genomic vRNA–minigenome and antigenomic cRNA–minigenome synthesis and evaluated the association between SFTSV NSs and N, RdRp, or viral RNA. Our findings not only provide the details of SFTSV NSs participating in viral RNA transcription and replication by using reverse genetics systems, but also contribute to better understanding of the multiple roles of SFTSV NSs in the virus replication cycle.

## Materials and Methods

### Cells and Virus

BHK-21 (baby hamster kidney) cells (CCL-10, ATCC; United States) were grown in Dulbecco’s modified Eagle’s medium (DMEM; GIBCO, United States) supplemented with 10% fetal bovine serum (FBS) at 37°C with 5% CO_2_. Hela (CCL-2, ATCC; United States) and Vero E6 (African green monkey kidney) cells (CRL-1586, ATCC; United States) were grown in Eagle’s Minimal Essential Medium (EMEM; GIBCO, United States) supplemented with 10% fetal bovine serum (FBS) at 37°C with 5% CO_2_. SFTSV (WCH97 strain) was grown in Vero E6 cells and handled in a biosafety level 3 laboratory as previously described (Ning et al., 2015, 2019; Feng et al., 2019).

### Plasmid Construction

To generate the minigenome transcription plasmids, pRF42 containing the murine pol I promoter and terminator was used as the backbone vector (Flick and Pettersson, 2001). For minigenome reporter plasmids, firefly luc or *EGFP* reporter genes flanked by viral UTR sequences were amplified by PCR and cloned into pRF42 in antisense orientation using the restriction-free clone method with an In-Fusion HD Clone kit (Clontech, Japan). The plasmid pRF42-luc (−)/(+) containing the antisense or sense reporter genes (firefly luc) not flanked by the viral UTR sequence was used as the control.

The helper plasmids of the minigenome reporter system, which encode SFTSV N (pCAG-SV-N), RdRp (pCAG-SV-RdRp), and NSs encoding plasmid pCAG-SV-NSs, were constructed by cloning the corresponding cDNA fragments into the expression vector pCAGGSP7 using double restriction enzyme (*Kpn*I and *Not*I) digestion and DNA ligation. Mutant NS plasmid was also constructed by cloning the mutant ORF of NS encoding gene with two proline residues (aa 66 and aa 69) changing to alanine into the expression vector pCAGGSP7 using double restriction enzyme (*Kpn*I and *Not*I) digestion and DNA ligation. The GenBank accession numbers of the viral segment reference sequences involved in the cloning of this study are as follows: JQ341188.1 (SFTSV L), JQ341189.1 (SFTSV M), and JQ341190.1 (SFTSV S); NC_024495.1 (HRTV L), NC_024494.1 (HRTV M), and NC_024496.1 (HRTV S). All cloning constructs were confirmed using sequencing.

### Antibodies

As previously described (Ning et al., 2015, 2017), rabbit anti-SFTSV NSs, NP, and RdRp or HRTV-NSs antiserum were respectively, raised against the corresponding viral proteins generated by *Escherichia coli*. The antibody anti-β-actin (ABclonal, China) was purchased from the manufacturer. For the secondary antibodies, goat anti-rabbit IgG conjugated with Alexa Fluor 488 (Thermo Fisher Scientific, United States) and goat anti-rabbit IgG conjugated with Alexa Fluor 555 (Thermo Fisher Scientific, United States) used in the IFA assay were purchased from the manufacturer; goat anti-rabbit IgG antibodies conjugated with HRP (Abcam, United States) were used for Western blot and Co-IP analysis.

### Minigenome Reporter Assays

BHK-21 cells cultured in 12-well plates were co-transfected with the indicated minigenome transcription plasmid (1.0 μg), RdRp expression plasmid (pCAG-SV-RdRp; 500 ng), N expression plasmid (pCAG-SV-N; 500 ng), and Renilla luciferase control plasmid (pRL-TK; 10 ng) per well using the Lipofectamine 3000 reagent (Invitrogen, United States) following the manufacturer’s instructions. In the *EGFP* system, the minigenome-luc reporter plasmid was replaced by the corresponding minigenome-*EGFP* plasmid, with the control plasmid pRL-TK omitted. After transfection for 48 h, cells were delivered to luc activity measurement using a dual-luciferase reporter kit (Promega, United States), and the firefly and Renilla luciferase activities (Luc. Act.) were measured as described previously (Ning et al., 2015, 2017). In the *EGFP* reporter system, *EGFP* expression was visualized under fluorescence microscopy.

### Protein–Protein Co-immunoprecipitation Assays

Lysates of the co-transfected or virus-infected cells were incubated with the specific antibodies at room temperature for 30 min and precipitated with protein A/G magnetic beads (MCE, United States). After incubation for 2 h, the beads were firstly washed four times with the binding buffer (PBST: 1 × PBS + 0.5% Triton X-100 at pH 7.4) and eluted with elution buffer (0.15 M glycine, 0.5% Triton X-100, or Tween-20 at pH 2.5–3.1). The immunoprecipitates were subjected to sodium dodecyl sulfate–polyacrylamide gel electrophoresis (SDS-PAGE), transferred onto PVDF membranes (Millipore, United States), and incubated with primary antibodies at a dilution of 1:2,000 at room temperature for 2 h and horseradish peroxidase (HRP)-conjugated secondary antibodies at a dilution of 1:2,000 for 2 h at room temperature. Signals on blots were developed by ECL reagents (Invitrogen, United States).

### Immunofluorescence Assay

BHK-21 or Hela cells transfected with plasmids encoding NSs or mutant NSs were washed twice with PBS, fixed with 4% paraformaldehyde for 20 min, permeabilized with 0.1% Triton X-100 for 10 min, and washed thrice with PBS. The cells were blocked with 5% bovine serum albumin in PBS for 1 h at 37°C and incubated with anti-NSs antibodies at 1:2,000 dilution at 37°C for 1 h. After three washes with PBST, the cells were incubated with goat anti-rabbit IgG (H + L) Highly Cross-Adsorbed Secondary Antibody (Alexa Fluor Plus 488, 1:1,000; Invitrogen, United States) at 37°C for 1 h and then washed and stained for 5 min with Hoechst 33342 (Invitrogen, United States). After washing, the cells were visualized under an EVOS FL Auto confocal microscope (Invitrogen, United States).

### Immunoblot Analysis

To ensure that NSs, N, and RdRp are expressed within the minigenome reporter systems as indicated on BHK-21 and Hela cells, Western blotting was performed with specific antibodies (anti-NSs, anti-N, or anti-RdRp antibodies) by using β-actin as an internal control. Briefly, equal amounts of SDS-loading buffer treated cell lysates of the transfected cells were subjected to SDS-PAGE, transferred onto PVDF membrane (Millipore, United States), and incubated with primary antibodies, rabbit-derived anti-N/NSs/RdRp, or mouse-derived anti-β-actin antibodies (Thermo Fisher Scientific, United States), at room temperature for 2 h. They were further incubated with HRP-conjugated secondary antibodies for 2 h at room temperature. Signals on blots were developed by ECL reagents (Invitrogen, United States).

### Protein–RNA Co-immunoprecipitation Assays

Immunoprecipitation combined with RT-PCR was conducted to detect virus-like RNA in immunoprecipitates. Briefly, cell lysates of the transfected or co-transfected cells were incubated with specific anti-NSs antibodies overnight at 4°C with an RNase inhibitor (Takara, Japan) and precipitated with protein A/G magnetic beads (MCE, United States). After 2-h incubation at 4°C, the beads were washed four times with the binding buffer and eluted with the elution buffer. Viral RNA was isolated from the immunoprecipitates with the TRIzol reagent (Invitrogen, United States) and subjected to RT-PCR. Primers used to amplify L/M/SUTR-Luc are as follows: LUTR-Luc, forward primer 5′-acacaaagaccgcccag-3′, reverse primer 5′-cttcacgttctctggcc-3′; MUTR-Luc, forward primer 5′-acacaaagaccggccaaand-3′, reverse primer 5′-ggccaacaatgatgaaa-3′; SUTR-Luc: forward primer 5′-acacaaagacccccttc-3′, reverse primer 5′-aggaaagacgcaaagga-3′. Primers used to amplify 2,000 bp of viral L, M, and S are as follows: L, forward primer 5′-ttaaccccacatttctg-3′, reverse primer 5′-ttgcttcaggtacactg-3′; M, forward primer 5′-ctaagccagctttgtcc-3′, reverse primer 5′-tcaaaggggcattggta-3′; S, forward primer 5′-atgtcgctgagcaaatg-3′, reverse primer 5′-atgtcagagtggtccag-3′.

### Virus Titration by Immunofluorescence Assay (IFA)

To analyze whether the overexpressed wild-type or mutant NSs could affect virus replication, we performed IFA to determine and compare the titer of virus supernatants collected at various times. Briefly, BHK-21 or Hela cells cultured in 12-well plates were transfected with 0.5, 1.0, and 2.0 μg of NSs expression plasmid DNA. After 12 h, the cells were infected with SFTSV at a multiplicity of infection (MOI) of 1.0. Supernatants were harvested at 12, 24, 36, 48, 72, 96, and 120 h post infection (hpi). Vero E6 cells (1 × 10^5^ cells/well) were seeded on 96-well plates and incubated for 24 h at 37°C in 5% CO_2_ to produce a confluent monolayer, inoculated with serial 10-fold dilutions of the virus supernatants obtained above, and titrated for infectious virus titration (TCID_50_) using indirect immunofluorescence as described previously (Ren et al., 2020). Infectious virus titers (TCID_50_/ml) were calculated based on the Reed and Muench method.

### Superinfection Assays

To further reveal the effect of NSs on viral RNA synthesis under infection. We adopted superinfection assays as previously described to compare the levels of viral RNA synthesis through analyzing the luciferase activities of minigenome (−) and minigenome (+) with overexpressed NSs or mutant NSs (Ren et al., 2020). Briefly, BHK-21 cells cultured in 12-well plates were transfected with 2.0 μg of NSs- or mutant NSs-expression plasmid DNA and 1.0 μg of L/M/S (−)/(+) minigenome plasmid; at 12 hpt, the cells above were infected with SFTSV at a MOI of 3.0, respectively, and the luciferase activities of minigenome (−) and minigenome (+) were measured at 48 hpi.

### Real-Time Quantitative PCR (RT-qPCR)

To measure the RNA levels of the indicated L, M, and S minigenome RNA, the total intracellular RNA content was extracted from cells transfected with minigenome plasmids using TRIzol reagent (Takara, Japan), and the first strand cDNA was synthesized by using the PrimeScript^TM^ RT reagent kit and quantitated by qPCR using TB Green^®^ Premix Ex Taq^TM^ II (Takara, Japan) as previously described (Ning et al., 2015; Mo et al., 2020). The data shown represent the relative abundance of the indicated RNA normalized to that of GAPDH. The primer sequences for GAPDH and SFTSV L, M, and S minigenome RNA were as follows: GAPDH: 5′-ACCACAGTCCATGCCATCAC-3′ (forward) and 5′-TCCACCACCCTGTTGCTGTA-3′ (reverse); L, M, and S minigenome RNA: 5′-GCCGCAGTTCCAGGAACACTA-3′ (forward) and 5′-GCAATGAGTTTCTAGATGTAA-3′ (reverse). All RT-qPCR experiments were performed on an ABI 7500 system according to the manufacturer’s instructions.

### Statistical Analysis

Statistical analyses were accomplished by GraphPad Prism 8, and data were analyzed using one-way analysis of variance (ANOVA). All data are presented as the mean ± SEM. *p* < 0.05 was considered statistically significant.

## Results

### The cDNA-Derived Virus-Like RNA Synthesis Systems Consisting of Genomic and Antigenomic Minigenomes of SFTSV Were Constructed

**FIGURE 1:**
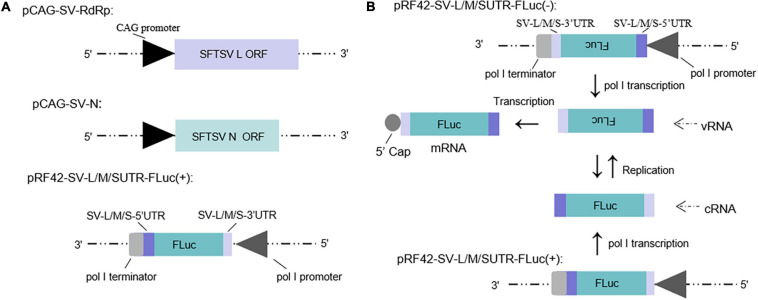
Schematic of the experimental generation of the pol I-driven SFTSV minigenome system and the cDNA-derived virus-like RNA synthesis system. **(A)** Organization of murine pol I-driven SFTSV L, M, and S minigenome systems. The minigenome system consists of the minigenome plasmid and the expression plasmids of RdRp and N. Briefly, ORF of SFTSV RdRp or N was respectively, inserted into vector pCAGGSP7 by double digestion (*Kpn*I and *Not*I); Firefly luciferase (FLuc) gene flanked by the 5′ and 3′ UTR of L/M/S was inserted into the pRF42 in antigenome (+) orientation. **(B)** Transcription and replication in the cDNA-derived virus-like RNA synthesis system of SFTSV. Virus-like vRNAs and cRNAs were respectively, transcribed by pRF42-SV-L/M/SUTR-FLuc (–) and pRF42-SV-L/M/SUTR-FLuc (+) based on murine pol I promoter. Meanwhile, cRNAs can be synthesized by replication using vRNAs as template and subsequently can be used as templates to synthesize vRNA by transcription. The vRNA can also be used as templates to synthesize mRNA through transcription. The 5′ capped mRNA can encode the reporter protein (FLuc or *EGFP*), which can be used as an indicator for viral RNA synthesis.

Minigenome (−) of SFTSV driven by murine pol I has been described in our previous study (Ren et al., 2020). The minigenome (+) system constructed in this work consists of SFTSV RdRp and N expression plasmids and the reporter plasmid that was inserted with the antisense orientation ORF of firefly luciferase (luc) or enhanced green fluorescent protein (*EGFP*) cassette flanked by the UTRs of SFTSV L, M, and S segments, respectively, to develop the minigenome (+) system (see section “Materials and Methods”) (Figure 1A).

The minigenome (−)/(+) generated the initial virus-like genome or anti-genome RNA (vRNA or cRNA), respectively, and completely simulated transcription and replication during infection. In the two systems, virus-like RNA synthesis, including replication and transcription, can occur, resulting in the expression of the reporter protein, which can be used as an indicator of the RNA synthesis level (Figure 1B). It is of significance to note that the number of vRNA-minigenome templates available for use in transcription is dependent on virus-like genome replication. An increase in virus-like genome replication leads to an increase in templates available for transcription, and thus, can lead to an increase in both mRNA levels and reporter activity. Therefore, the reporter activity as well as the mRNA amount reflects not only viral transcription but also viral genome replication in the minigenome systems we used.

### SFTSV NSs Decreases the Reporter Activity Within Minigenome (−)/(+) Systems

To investigate the role of NSs in virus-like RNA synthesis within the minigenome systems, we exploited multi-plasmid co-transfection combined with firefly luciferase activity determination assays to accurately quantify altered efficiencies in viral RNA synthesis levels.

**FIGURE 2:**
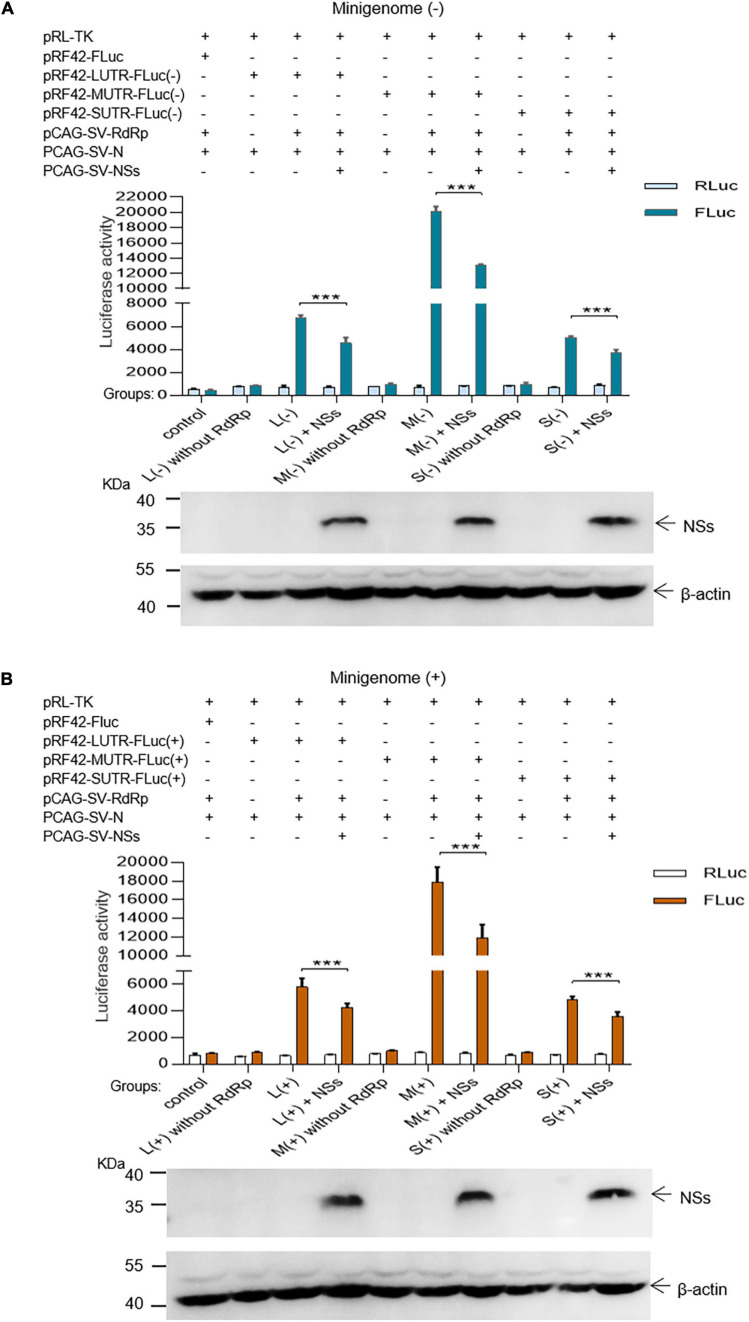
SFTSV NSs decrease the minigenome (–)/(+)-encoded reporter activity. NSs downregulated reporter activity within the minigenome (–) and minigenome (+) systems based on viral L, M, and S denoted as L (–)/(+), M (–)/(+), and S (–)/(+), respectively. Briefly, groups of various plasmid mixes indicated in **(A,B)** were transfected into BHK-21 cells cultured in 12-well plates with Lipo 3000, respectively. The symbol “+” means the presence of designated plasmid in plasmid mixes and “–” means the absence of designated plasmid in plasmid mixes. Cells were transfected with 1.0 μg of minigenome (+)/(–) plasmids or control plasmids pRF42-FLuc, 0.5 μg RdRp, N, and NSs expression plasmids, along with 10 ng of internal control plasmid pRL-TK to express Renilla luciferase. Meanwhile, negative control groups including L (–)/M (–)/S (–) without RdRp and L (+)/M (+)/S (+) without RdRp were independently set up to check that the integrity of the minigenome system is important for virus-like RNA synthesis in both minigenome (–) and minigenome (+) systems. At 48 hpt, firefly luciferase and Renilla luciferase were measured by using Dual-Glo Luciferase Assay System kit as the technical manual and data are presented as the mean ± standard error of mean (SEM), *n* = 3. ***p < 0.001 **(A,B)**. Meanwhile, the NSs expression levels were analyzed by Western blotting by using β-actin as internal control **(A,B)**.

In the L, M, and S segment-based SFTSV minigenome (−)/(+) systems, functional RNPs efficiently assembled to express reporter protein compared with the groups where RdRp was omitted and negative controls. Luciferase activations (Luc. Act.) for L (−), M (−), S (−), L (+), M (+), and S (+) decreased significantly when NSs encoding plasmids (0.5 μg for each test) were co-transfected (Figures 2A,B), and they decreased by 32.3, 34.9, 26.2, 26.0, 33.6, and 27.3%, respectively. These results suggest that the NSs downregulated virus-like RNA synthesis in both minigenome (−) and minigenome (+) systems, but not in the manner of significant difference for the two systems.

### SFTSV NSs Downregulate Virus-Like RNA Synthesis in a Dose-Dependent Manner

As the initial cDNA-derived product is vRNA for the minigenome (−) system, the genomic minigenome is more suitable than the antigenomic minigenome to mimic authentic viral RNA synthesis in mammalian cells (Kolakofsky and Hacker, 1991; Guu et al., 2012). We next investigated whether SFTSV NSs downregulate the virus-like RNA synthesis in a dose-dependent manner using the minigenome (−) system.

**FIGURE 3:**
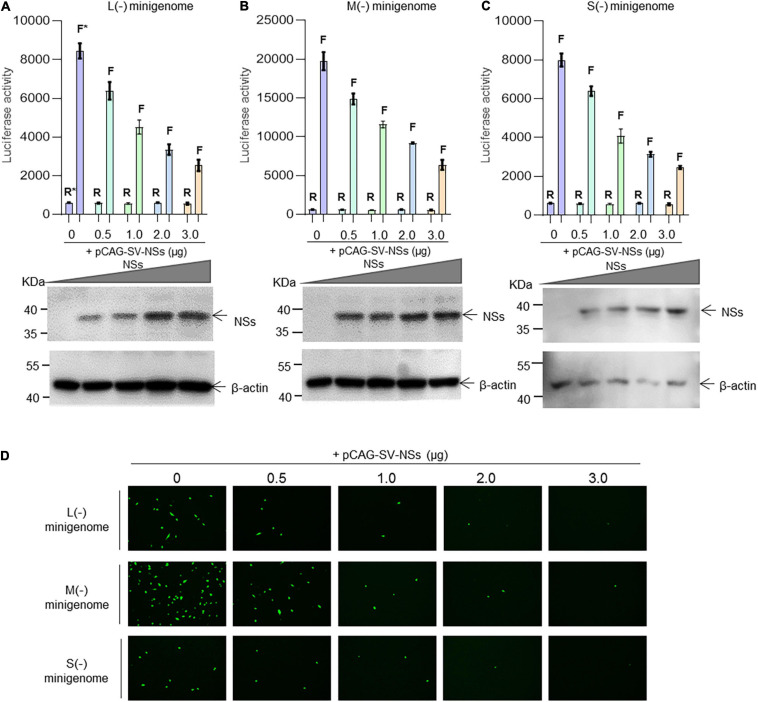
SFTSV NSs downregulate virus-like RNA synthesis in a dose-dependent manner. We adopted a minigenome (–) reporter system combined with Western blotting to determine whether the regulatory effect is dose-dependent. BHK-21 cells cultured in 12-well plates were transfected with 1.0 μg of minigenome (–) plasmids (pRF42-L/M/SUTR-FLuc or pRF42-L/M/SUTR-EGFP) or control plasmid pRF42-FLuc, 0.5 μg of RdRp, N, and NSs expression plasmids, along with 10 ng of internal control plasmid pRL-TK (omitted for *EGFP* reporter system) to express Renilla luciferase. The activity of firefly luciferase and Renilla luciferase was measured, and data for L (–) (**A**, top), M (–) (**B**, top), and S (–) (**C**, top) are presented as the mean ± SEM, *n* = 3. Meanwhile, the cell lysates of L (–) (**A**, bottom), M (–) (**B**, bottom), and S (–) (**C**, bottom) were subjected to Western blotting analysis with β-actin as an internal loading control. In the *EGFP* reporter system, cells were observed under fluorescence microscopy **(D)**. In **(A–C)**, the symbols “R” and “F” (marked with * in **A**) above the indicated bars represent Renilla luciferase activation and firefly luciferase activation, respectively.

As shown in Figure 3, when the NSs-expression plasmids were added to the mixed plasmids of the minigenome systems, the L (−), M (−), and S (−) minigenomes all showed decreased viral RNA synthesis levels compared with the corresponding minigenome systems without the involvement of NSs. In the L/M/S (−) minigenome, upon the addition of NSs-expression plasmid at 0.5, 1.0, 2.0, and 3.0 μg (Figures 3A–C, bottom), the levels of viral RNA synthesis decreased significantly as evidenced by the decreased luciferase activity, respectively, of 24.5, 46.6, 60.3, and 70.0% for L (−) compared with the control group (Figure 3A) of 24.7, 41.3, 53.5, and 67.8% for the M (−) minigenome (Figure 3B) and of 20.0, 48.9, 60.8, and 69.3% for the S (−) minigenome (Figure 3C). Similar results were observed from the *EGFP*-reporter systems in that the *EGFP* signals reduced upon the increase of co-transfected NS plasmid in the L (−), M (−), and S (−) minigenome systems (Figure 3D).

### SFTSV NSs Are Associated With N but Not RdRp Within the Minigenome Reporter Systems and Virus-Infected Cells

Since the NSs downregulate the virus-like RNA synthesis within minigenome reporter systems, it would be urgent to elucidate the possible mechanism of how NS protein regulates viral RNA synthesis. Here, we utilized protein–protein/RNA co-immunoprecipitation and RT-PCR assays to dissect what components of SFTSV RNP are associated with the NSs within L (−), M (−), and S (−) minigenome systems and virus-infected cells.

**FIGURE 4:**
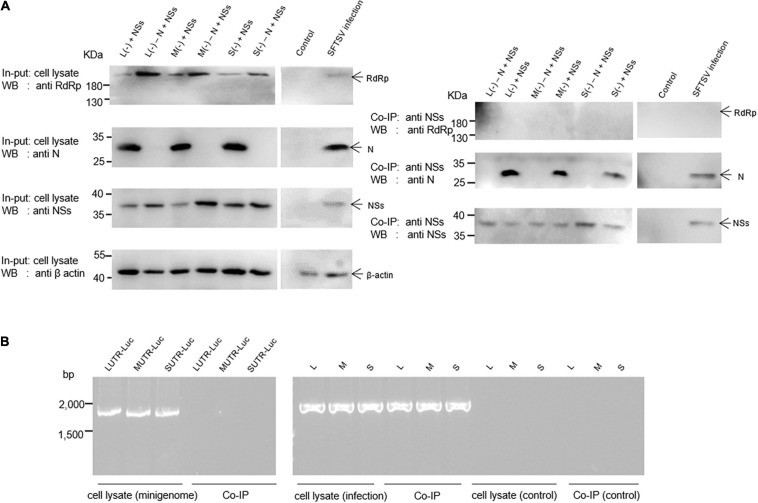
SFTSV NSs are associated with N but not RdRp within the minigenome reporter systems and virus-infected cells. Co-IP assays and RT-PCR were used to identify the viral components interacting with NSs. Minigenome assays was carried out as previously described, and the amounts of expression plasmid and transcription plasmid were 0.5 μg and 1.0 μg, respectively. The amount of NSs expression plasmids was 1.0 μg. Meanwhile, L (–), M (–), and S (–) that do not contain N expression plasmid were set up as negative controls, denoted as L (–)/M (–)/S (–) – N + NSs, respectively. At 48 hpt, cell lysates of L (–)-, M (–)-, and S (–)-transfected and virus-infected cells were subjected to Western blotting analysis (**A**, left) and Co-IP (**A**, right), respectively. Meanwhile, cell lysates and co-immunoprecipitates of L (–)-, M (–)-, and S (–)-transfected and virus-infected or control cells were all treated with TRIzol reagent to extract virus-like and viral RNA, which were then subjected to RT-PCR analysis **(B)**.

The results indicate that when BHK-21 cells were co-transfected with the plasmids of the L/M/S (−) minigenome system comprising pCAG-SV-N, pCAG-SV-RdRp, pRF42-L/M/SUTR-luc (−), and NSs-expression plasmid pCAG-SV-NSs or were infected with SFTSV, viral proteins, including RdRp, N, and NSs, were detected in the cell lysates by Western blotting (Figure 4A, left). To reveal the viral components associated with the NSs, we co-immunoprecipitated the designated cell lysates with anti-NSs antibodies by using Protein A/G Magnetic Beads kits (MCE, United States) and then the immunoprecipitates were subjected to Western blotting analyses. Meanwhile, the viral RNA was extracted from cell lysate and immunoprecipitates with TRIzol reagent and then was subjected to RT-PCR analysis. Our results suggest that NSs are associated with N, not with RdRp (Figure 4A, right). Intriguingly, the amount of N in the immunoprecipitates increased with the amount of NSs (data not shown), further supporting the interaction between NSs and N. Viral L-, M-, or S-like RNA (denoted as LUTR-luc, MUTR-luc, and SUTR-luc, respectively) was only detected in the cell lysates but not in co-immunoprecipitates (Figure 4B). However, Viral L, M, or S could be obviously detected both in the cell lysates and co-immunoprecipitates but not in the control group (Figure 4B).

### The Negative Regulatory Effect of NSs on Virus-Like RNA Synthesis Could Be Outcompeted by Increasing the Expression Levels of N

**FIGURE 5:**
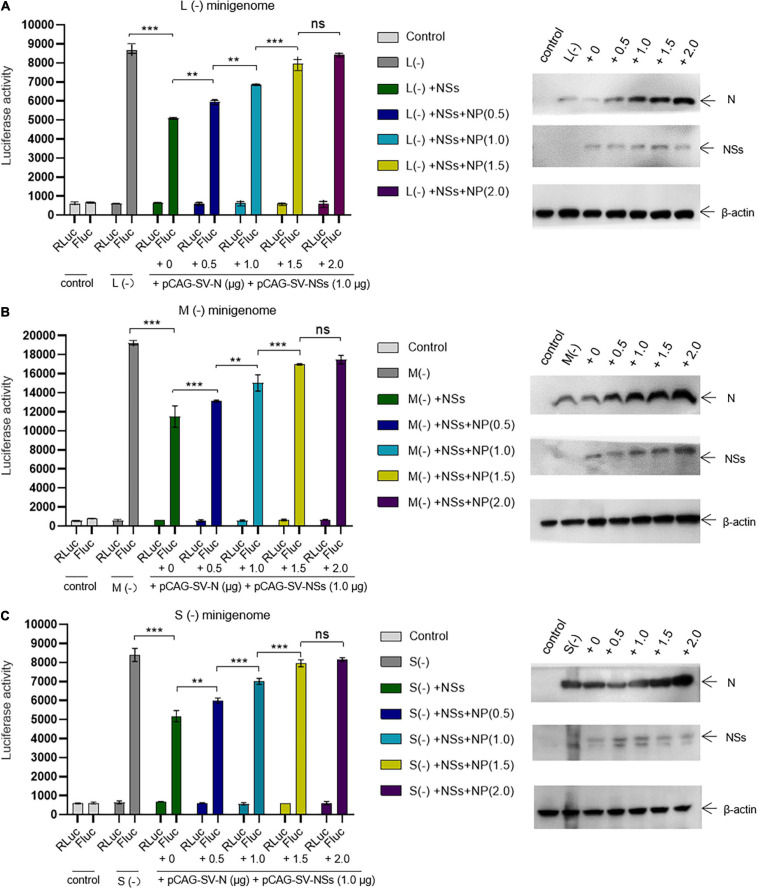
The negative regulatory effect of NSs on virus-like RNA synthesis can be outcompeted by increasing the level of N. Minigenome reporter assays combined with Western blotting analysis was carried out to investigate whether the accumulation of N reverses the negative regulatory effect on virus-like RNA synthesis. Minigenome reporter assays were carried out as described above. Briefly, BHK-21 cells cultured in 12-well plates were transfected with 1.0 μg of pRF42-L/M/SUTR-FLuc (–), 0.5 μg of pCAG-SV-RdRp, and 0.5 μg of pCAG-SV-N together with 10 ng of internal control plasmid (pRL-TK). Meanwhile, control groups with pRF42-L/M/SUTR-FLuc (–) being replaced with pRF42-FLuc were set, respectively. Moreover, cells transfected with plasmid mixes of L (–), M (–), and S (–) with 0.5 μg of NSs protein expression plasmids pCAG-SV-NSs are also transfected with various amount of additional N expression plasmids pCAG-SV-N (0, 0.5, 1.0, 1.5, and 2.0 μg, respectively). After 48 h, firefly and Renilla luciferase were measured. Data are presented as the mean ± SEM (*n* = 3). **p < 0.01; ***p < 0.001; ns, non-significant (**A–C**, left). To confirm the expression of NSs and the accumulation of N, Western blotting was conducted (**A–C**, right).

We next assessed whether the inhibitory effect of NSs on virus-like RNA synthesis could be reversed. We found that when the amount of transfected N-expression plasmid DNA increased, the inhibitory effect of NSs on L, M, and S virus-like RNA synthesis decreased. In the L/M/S (−) minigenome system, the activations of firefly luciferase decreased significantly with the presence of overexpressed NSs. When the amount of additional N-expression plasmid DNA was increased sequentially from 0 to 0.5, 1.0, and 2.0 μg (Figures 5A–C, right), the activations of firefly luciferase (Luc. Act.) were restored significantly compared with the control groups (Figures 5A–C, left). The Luc. Act. increased by 16.8, 34.9, 56.8, and 65.8%, respectively, for the L (−) minigenome system (Figure 5A, left). Similarly, in the M (−) minigenome system, the activations of firefly luciferase increased by 14.1, 30.7, 47.5, and 51.9%, respectively (Figure 5B, left), and in the S (−) minigenome system, the activations of firefly luciferase increased by 15.7, 35.1, 53.5, and 57.2%, respectively (Figure 5C, left). In contrast, when the amount of RdRp-expression plasmid DNA increased, the inhibitory effect of NSs did not significantly change (see Supplementary Figure 1). These results showed that NSs exert negative regulatory effects on virus-like RNA synthesis and that the accumulation of N but not RdRp can reverse the inhibitory effect.

### Formation of IBs Induced by NSs Is Associated With Its Inhibitory Effect on Virus-Like RNA Synthesis

**FIGURE 6:**
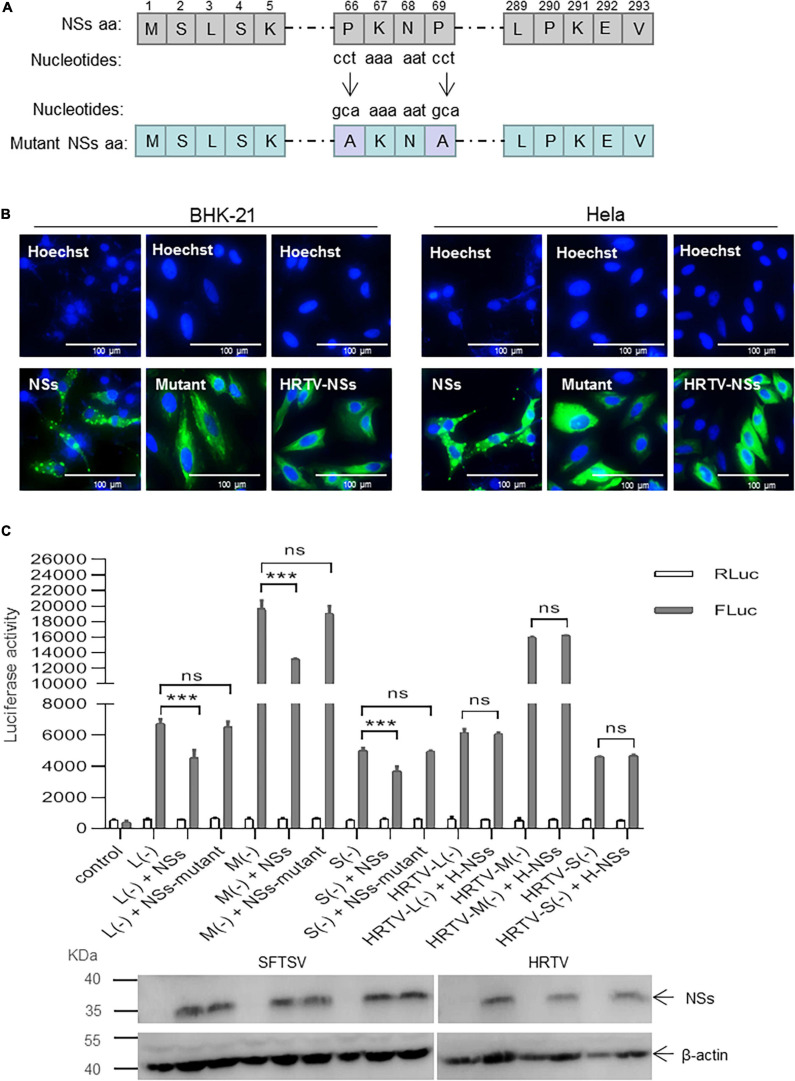
Formation of NSs-induced IB structure is important for the suppression of virus-like RNA synthesis. **(A)** Schematic of mutant NSs clone. The amino acids at positions 66 and 69 were substituted by alanine *via* the introduction of mutant nucleotides to NSs encoding gene (“cct” were mutated to “gca”). Mutant clone (pCAG-SV-NSs-mutant) was constructed by double restriction enzyme digest (*Kpn*I and *Not*I) and In-Fusion technology (Clontech, Japan). **(B)** When BHK-21 and Hela cells were respectively, transfected with pCAG-SV-NSs, pCAG-SV-NSs-mutant, or pCAG-HRTV-NSs, IFA was performed 48 hpt to observe wild-type, mutated SFTV NSs and HRTV NSs under immunofluorescence microscopy. **(C)** Minigenome reporter assays were performed as previously described. BHK-21 cells in a 12-well plate were transfected with 1.0 μg of pRF42-SV/HRTV-L/M/SUTR-FLuc (–), 0.5 μg of pCAG-SV-RdRp, and 0.5 μg of pCAG-SV-N together with 0.5 μg of SFTSV NSs/mutant NSs-expression plasmid DNA or HRTV NSs-expression plasmid DNA and 10 ng of internal control plasmid pRL-TK. Meanwhile, a control group with pRF42-FLuc replacing pRF42-SV/HRTV-L/M/SUTR-FLuc was also set up. The activities of firefly and Renilla luciferase were measured at 48 hpt, respectively. Data are presented as the mean ± SEM (*n* = 3). ***p < 0.001; ns, non-significant. The expression levels of SFTSV NSs/mutant NSs and HRTV NSs were analyzed, respectively, by Western blot.

To investigate whether the interaction between NSs and N is associated with the IB formed by NSs, we introduced the mutant NSs in which two prolines at positions of 66 and 69 were mutated to alanines (designated as mutant) by site-directed mutagenesis (Figure 6A). As shown in Figure 6B, when the two prolines at positions 66 and 69 were mutated to alanine, the structure of IB formed by the NSs can hardly be observed compared with the wild-type NSs in both BHK-21 and Hela cells. Meanwhile, HRTV NSs that cannot form IB structure in the cytoplasm was also introduced as a comparison (Figure 6C).

When BHK-21 cells were transfected with the same amount (0.5 μg) of plasmid DNA encoding SFTSV NSs/mutant NSs or HRTV NSs together with mixed plasmids of the minigenome reporter system and internal control plasmid pRL-TK, there was no significant difference in firefly luciferase activation in the L (−), M (−), and S (−) minigenome systems with mutant NSs, whereas with wild-type NSs, the firefly luciferase activations decreased significantly (Figure 6C). However, the NSs of HRTV exerted no significant activity-inhibition or -promotion effect on HRTV L, M, and S minigenomes (−) (Figure 6C). These results suggest that the suppressive effect of NSs on virus-like RNA synthesis is due to its interaction with N, and the interaction is closely associated with the structure of IB formed by NSs.

### NSs Exert a Negative Effect on Viral RNA Synthesis and Virus Replication in Immunodeficient and Non-immunodeficient Cells

To further reveal the role of NSs in authentic virus replication and the potential correlation between NSs regulating viral RNA synthesis and antagonizing the host’s antiviral responses, we adopted transfection combined with the superinfection method to compare the efficiencies of virus-like RNA synthesis and virus replication in immunodeficient (BHK-21) and non-immunodeficient (Hela) cells (Andzhaparidze et al., 1981; Matskevich et al., 2009), respectively.

**FIGURE 7:**
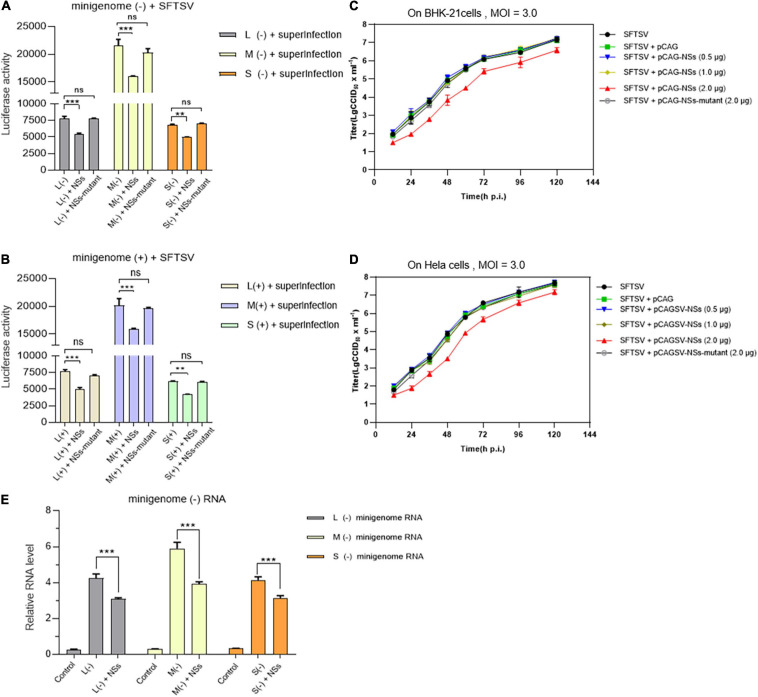
NSs exert a negative regulatory effect on virus-like RNA synthesis and virus replication in both immunodeficient cells and non-immunodeficient cells. To further investigate the role of overexpressed NSs in virus-like RNA synthesis, virus replication, and the correlation between NSs regulating RNA synthesis and antagonizing host antiviral responses, we adopted superinfection and infection methods to analyze virus-like RNA synthesis and virus replication under different conditions. Briefly, when BHK-21 cells transfected with 1.0 μg of L/M/S minigenome (–) or minigenome (+) and 2.0 μg of NSs- or mutant NSs-expression plasmids were infected with SFTSV at a MOI of 3.0, the luciferase activity (Luc. Act.) of the indicated groups were measured at 48 hpi, respectively. Data are presented as the mean ± SEM (*n* = 3) for minigenome (–) **(A)** and minigenome (+) **(B)**. **p < 0.01; ***p < 0.001; ns, non-significant. The immunodeficient (BHK-21) or non-immunodeficient cells (Hela) were transfected with various amounts (0.5, 1.0, and 2.0 μg) of NSs- or mutant NSs-expression plasmids DNA, respectively. At 12 hpi, the indicated cells above were infected with SFTSV at a MOI of 3.0, respectively, and the supernatants were harvested at 12, 24, 36, 48, 72, 96, and 120 hpi. The supernatants then were titrated for infectious virus titration (TCID_50_) using indirect immunofluorescence assays. Infectious virus titers (TCID_50_/ml) were calculated based on the Reed and Muench method. Data are presented as the mean ± SEM (*n* = 3) **(C,D)**. The minigenome RNA levels of L (–), M (–), and S (–) were quantified by qRT-PCR using the gene encoding GAPDH as an internal control and the relative RNA levels were presented **(E)**. In the qPCR analyses, relative RNA levels over the control groups (wild-type cells or the cells transfected with the control plasmids) were calculated for L (–), M (–), and S (–) minigenome RNA, respectively. Data show mean ± SEM (*n* = 3). ***p < 0.001.

As shown in Figures 7A,B, when BHK-21 cells transfected with L/M/S minigenome (−)/(+) and NSs-expression plasmid were infected with SFTSV at a MOI of 3.0, the levels of virus-like RNA synthesis decreased significantly for both minigenome (−) and minigenome (+) by measuring and comparing the luciferase activities, while similar changes were not observed when the NSs-expression plasmid was replaced with the NSs-mutant-expression plasmid. As shown in Figures 7C,D, when immunodeficient BHK-21 cells and non-immunodeficient Hela cells were infected with SFTSV at a MOI of 3.0, there were no obvious differences in the one-step growth curves of the cells when the amount of transfected NSs-expression plasmid DNA was 0.5 and 1.0 μg, respectively; upon the transfection of 2.0 μg NSs-expression plasmid DNA, the virus titers were lower than the control groups at various time points. However, when the NSs-expression plasmid was replaced with the plasmid DNA encoding mutant NSs, there were no significant differences in the titers at various time points compared with the control group. Meanwhile, the RT-qPCR results showed that the NSs can directly decrease the synthesis of L-, M-, and S-minigenome RNA significantly compared with the controls (Figure 7E). These results indicate that low levels of overexpressed NSs and mutant NSs do not affect SFTSV replication on both BHK-21 and Hela cells and it seems that there are no correlations between regulating viral RNA synthesis and antagonizing antiviral innate immunity by NSs.

## Discussion

Reverse genetics systems have been utilized to study the roles of virus components played in the viral RNA replication and transcription. In a typical minigenome system, the minigenome transcripts expressed by either host RNA pol I or T7 RNA polymerase contain an internal ORF of a reporter gene instead of a viral ORF flanked by viral UTRs (Hoenen et al., 2011). The expressed minigenome RNA transcripts undergo RNA replication and transcription in the presence of co-expressed viral proteins including RdRp and N, or co-infected helper virus. The levels of reporter expression indicate the efficiency of the minigenome RNA replication and transcription. The regulatory effect of NSs on viral RNA synthesis has been previously reported in many other bunyaviruses by using minigenome systems. For instance, Weber et al. (2001) have reported that the NSs derived from BUNV, Guaroa virus, and Lumbo virus inhibit virus-like RNA synthesis in T7-polymerase-driven minigenome systems by regulating the activity of the viral polymerase in a highly conserved mechanism. Similar to BUNV NSs, Blakqori et al. (2003) have reported that the NSs of La Crosse virus (LACV) can downregulate the activity of the minigenome, thus leading to the downregulation of virus-like RNA synthesis. On the contrary, the NSs of bunyaviruses may also exert a positive regulatory effect on viral RNA synthesis. Rift Valley fever virus (RVFV) NSs locating in both the cytoplasm and nucleus of infected cells can enhance virus-like RNA replication and transcription in a novel RVFV minigenome system (Ikegami et al., 2005). These suggest that the NSs of bunyaviruses adopt various strategies in regulating virus-like or viral RNA synthesis. Our results here showed that SFTSV NSs play a negative regulatory role in virus-like RNA synthesis and that the underlying mechanism of which is not associated with its previously identified role in counteracting antiviral innate immunity.

We have previously employed a cDNA-derived RNA synthesis system, i.e., minigenome (−), that generates RNA replication and transcription products to identify the elements affecting viral promoter activity (Ren et al., 2020). In this study, we utilized pol I-driven minigenome (−)/(+) systems to generate the virus-like RNAs, including vRNA and cRNA, involved in transcription and replication. Using these established systems, we found that SFTSV NSs encoded by the S segment downregulate vRNA- and cRNA-minigenome synthesis by sequestering N through blocking the interaction between N and naked transcripts of the minigenome (−)/(+) and the RNA derived from the intermediate process (cRNA or vRNA), while SFTSV NSs cannot seem to efficiently block the interaction between viral transcripts and N, which may be due to the sequence differences between virus-like and viral RNA (Figures 4A,B). Wu et al. (2014) have reported that SFTSV NSs are associated with N and viral S segments and may serve as a virus replication factory in infected cells. Similarly, in the pol I-driven minigenome systems, we found that NSs did interact with N; however, RdRp and virus-like RNA were not detected in the immunoprecipitates by NSs even though viral RNA could be detected (Figures 4A,B). Intriguingly, when BHK-21 or Hela cells transfected with low amounts of plasmids DNA (0.5 or 1.0 μg) expressing NSs were superinfected with SFTSV, NSs did not affect virus replication significantly (Figures 7C,D). According to the results of Figures 5A–C, we speculate that the accumulation of N due to virus replication reversed the inhibitory effect. However, when the transfected plasmid DNA expressing NSs was increased to 2.0 μg, the negative effect of NSs on virus replication was obviously observed, whereas the mutant NSs showed no regulatory effect. Meanwhile, we observed no cell apoptosis when the amount of NSs-expression plasmid increased to the maximum extent in this research through morphology analysis, which prove that the cytotoxicity effect of NSs on host cells was negligible. These results altogether indicate that the IB formed by NSs is critical for its regulatory role in virus-like RNA synthesis and virus replication.

**FIGURE 8:**
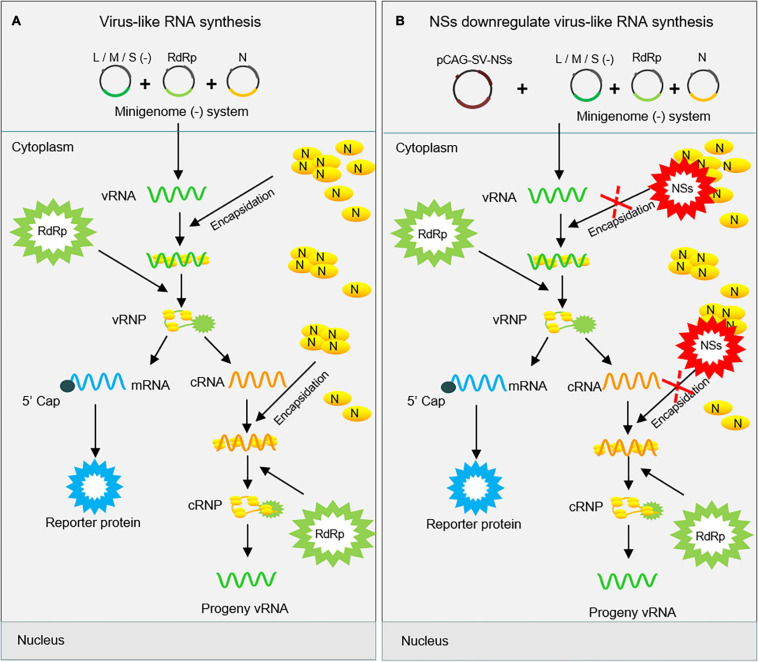
Proposed mechanism for the downregulation of virus-like RNA synthesis by SFTSV NSs within the minigenome reporter system. SFTSV NSs inhibit virus-like RNA synthesis by blocking the N–RNA interaction through sequestering N into inclusion bodies within minigenome reporter systems. As shown in **(A)**, initial genome virus-like RNA (vRNA) is generated by pol I transcription using cDNA as templates. After encapsidation by N, the coated nascent vRNA can assemble with RdRp to form vRNP. Then, mRNA and progeny cRNA and vRNA are generated by transcription and replication, respectively. However, overexpressed NSs block the encapsidation of viral RNAs by competing with nascent RNA to interact with N **(B)**. This, downregulates the synthesis of viral RNA, thus leading to decreased levels of the reporter protein.

Severe fever with thrombocytopenia syndrome virus NSs can form IBs by its own self in both virus-infected cells and NSs-expression plasmid-transfected cells (Wu et al., 2014; Moriyama et al., 2018). Previous studies have speculated on the possible mechanism underlying the viral immune evasion strategy adopted by SFTSV NSs that NSs can sequester critical signaling molecules from the mitochondrial antiviral platform through spatial isolation (Qu et al., 2012; Ning et al., 2014; Santiago et al., 2014). A clue that SFTSV NSs may be involved in virus-like and viral RNA synthesis has been provided by other researchers, though their results suggested that NSs seem to have exerted an opposite regulating effect on virus-like and viral RNA synthesis (Wu et al., 2014; Brennan et al., 2015). However, the details of SFTSV NSs regulating virus-like or viral RNA synthesis are not well elucidated yet. Here, we provide details that SFTSV NSs suppresses virus-like RNA synthesis through blocking the interaction between N and naked virus-like RNA by utilizing pol I-driven minigenome reporter systems and superinfection assays and thereby proposed a possible mechanism. As shown in Figure 8A, following the expression of viral RdRp, N, and naked minigenomes within the minigenome system, encapsidation will occur to reconstitute a viable RNP complex that can serve as a template for further transcription and replication. After the encapsidation of virus-like vRNA by N, the coated N can recruit RdRp to form functional vRNP, which can be transcribed into virus-like cRNA and mRNA. The mRNA can then be translated into reporter protein, which can be used to quantify the RNA synthesis level. Additionally, the N-coated virus-like cRNA can recruit RdRp to form functional cRNP, which can be transcribed into progeny virus-like vRNA. However, like the NSs of BUNV and LACV, our results showed that SFTSV NSs can decrease the virus-like RNA synthesis, resulting in decreased minigenome-encoded reporter activity, which can be presumably explained as the encapsidation of vRNA and cRNA were partially blocked *via* the NSs–N interaction (Figure 8B). Meanwhile, NSs exert a negative regulatory effect on SFTSV replication in both immunodeficient cells and non-immunodeficient cells, supporting that its inhibitory effect on virus-like RNA synthesis is not associated with NSs-mediated viral immune evasion. Interestingly, mutant SFTSV NSs cannot form IB, and it does not affect virus-like or viral RNA synthesis, and a similar effect is also detected in HRTV minigenome (−) systems with the involvement of HRTV NSs (Figures 6A–C). These results suggest that the inhibitory effect of SFTSV NSs on virus-like RNA synthesis and virus replication is caused by the NSs–N interaction, which may block the encapsidation of naked virus-like RNA.

Viral genomes of segmented negative RNA viruses are always assembled with many copies of a single nucleoprotein (N) to form highly stable nucleocapsids (Ruigrok et al., 2011; Sun et al., 2018), which is critical for transcription and replication. Similarly, in the case of a viral minigenome system based on plasmids or with a helper virus, encapsidation of naked minigenomes is indispensable for virus-like RNA transcription and replication. Although this step is particular to reverse genetics systems and has no strictly equivalent in the virus life cycle, it is of great significance to reveal the factors affecting the interaction between N and virus-like RNAs, especially when minigenome systems are used as tools to investigate viral genome transcription and replication, virus replication and pathogenesis. We here demonstrate the details that SFTSV NSs inhibit virus-like RNA synthesis through NSs–N interaction and in a dose-dependent manner. However, NSs of SFTSV seem to have little effect on virus replication until large amounts of overexpressed NSs were used, which can be presumably explained as the high-level expression of N by virus that can reverse the effect of NSs on virus replication to some extent. To further evaluate the effect of NSs on viral RNA synthesis or virus replication, it is crucial to rescue NSs deletant SFTSV using reverse genetics technology. It has been reported that a NSs deletant SFTSV was successfully rescued and the recombinant virus replicated more efficiently than wild-type virus in cells that had a defective interferon response (A549-NPro) but not in interferon competent cells (A549); meanwhile, their results also suggested that the NSs were not necessary for virus rescue or virus replication, even in cells with a functional IFN response (Brennan et al., 2017). These results consist of what we obtained by using minigenome reporter systems, revealing that the overexpressed NSs show a weak inhibitory effect on virus replication.

In conclusion, we have demonstrated that SFTSV NSs are involved in and inhibit virus-like RNA synthesis by using minigenome systems based on transfection or superinfection *via* a NSs–N interaction. In contrast, for virus replication or viral RNA synthesis, the effect is weak. Based on these results, we hypothesize that the NSs–N interaction partially blocked the encapsidation of naked virus-like RNA or viral RNA, resulting in decreased transcription and replication of minigenomes or the viral genome. The majority of bunyaviruses can encode NSs; although NSs in different bunyaviruses possess different amino acid sequences, the characterization of their common biological functions is important to better understand the life cycle of bunyavirus and to develop effective antiviral drugs and vaccines (Ly and Ikegami, 2016). With this in mind, further studies to reveal novel biological functions of NSs in emerging bunyaviruses are needed. For example, the roles in viral RNA synthesis for the NSs of other pathogenic bunyaviruses (Guertu virus and Bhanja virus, for instance), remain to be studied.

## Data Availability Statement

The original contributions presented in the study are included in the article/Supplementary Material, further inquiries can be directed to the corresponding authors.

## Author Contributions

FD, D-YZ, CH, and FR conceived and designed the study, analyzed the data, and wrote the manuscript. FR, SD, MZ, and JS performed the experiments and analyzed the data. HW, Y-JN, and QW helped with experimentation, data processing, and data analysis. FR and SS wrote the original manuscript. All authors contributed to the article and approved the submitted version.

## Conflict of Interest

The authors declare that the research was conducted in the absence of any commercial or financial relationships that could be construed as a potential conflict of interest.

## Publisher’s Note

All claims expressed in this article are solely those of the authors and do not necessarily represent those of their affiliated organizations, or those of the publisher, the editors and the reviewers. Any product that may be evaluated in this article, or claim that may be made by its manufacturer, is not guaranteed or endorsed by the publisher.
