# Supplementary material for: Non-structural Proteins of Severe Fever With Thrombocytopenia Syndrome Virus Suppress RNA Synthesis in a Transcriptionally Active cDNA-Derived Viral RNA Synthesis System
Source: Front Microbiol. 2021 Aug 16;12:709517. doi: 10.3389/fmicb.2021.709517 (PMC8415556; doi:10.3389/fmicb.2021.709517)
Supplement: Supplementary file 1 [file Data_Sheet_1.PDF]

Supplementary Material

Supplementary Figures

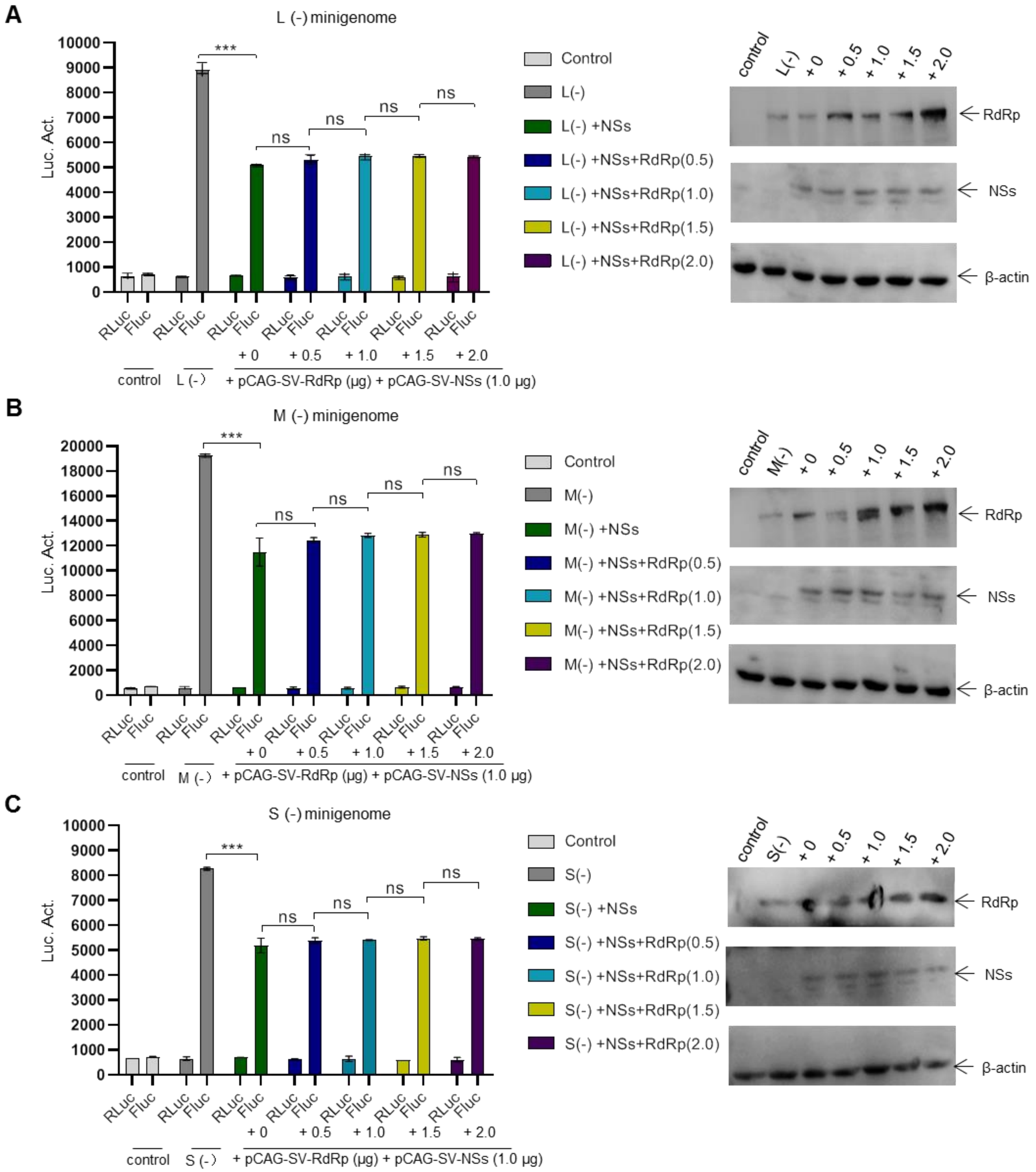

Supplementary Figure 1. The negative regulatory effect of NSs on virus-like RNA synthesis cannot be outcompeted by increasing the level of RdRp. Minigenome reporter assays combined with western blotting analysis was carried out to investigate whether the accumulation of RdRp reverses the negative regulatory effect on virus-like RNA synthesis. Minigenome reporter assays was carried out as described above. Briefly, BHK-21 cells cultured in 12-well plate were transfected with 1.0 μg pRF42-L / M / SUTR-FLuc (−), 0.5 μg pCAG-SV-RdRp, and 0.5 μg pCAG-SV-NP together with 10 ng internal control plasmid (pRL-TK). Meanwhile, control groups with pRF42-L / M / SUTR-FLuc (−) being replaced with pRF42-FLuc were set, respectively. Moreover, cells transfected with plasmid mixes of L (−), M (−) and S (−) with 0.5 μg NSs expression plasmids pCAG-SV-NSs are also transfected with various amount of additional RdRp expression plasmids pCAG-SV-RdRp (0, 0.5, 1.0, 1.5 and 2.0 μg, respectively). After 48 h, firefly and renilla luciferase were measured. Data are presented as the mean ± SEM (n = 3). ** $P < 0.01$; *** $P < 0.001$; ns, nonsignificant (as shown in **A**, **B**, and **C**, left). To confirm the expression of NSs and the accumulation of RdRp, western blotting was conducted (**A**, **B** and **C**, right).
